# A New Semi-automated Method for Assessing Avian Acoustic Networks Reveals that Juvenile and Adult Zebra Finches Have Separate Calling Networks

**DOI:** 10.3389/fpsyg.2016.01816

**Published:** 2016-11-29

**Authors:** Marie S. A. Fernandez, Hedi A. Soula, Mylene M. Mariette, Clémentine Vignal

**Affiliations:** ^1^Univ Lyon, UJM-Saint-Etienne, Centre Nationnal de la Recherche Scientifique, Neuro-PSI/ENES UMR9197Saint-Etienne, France; ^2^EPI BEAGLE INRIAVilleurbanne, France; ^3^Institut National de la Santé et de la Recherche Médicale U1060 INSAVilleurbanne, France; ^4^Centre for Integrative Ecology, School of Life and Environmental Sciences, Deakin UniversityGeelong, VIC, Australia

**Keywords:** development, ontogeny, songbird, acoustic communication, vocal interactions, turn-taking, conversation rules

## Abstract

Social networks are often inferred from spatial associations, but other parameters like acoustic communication are likely to play a central role in within group interactions. However, it is currently difficult to determine which individual initiates vocalizations, or who responds to whom. To this aim, we designed a method that allows analyzing group vocal network while controlling for spatial networks, by positioning each group member in equidistant individual cages and analyzing continuous vocal interactions semi-automatically. We applied this method to two types of zebra finch groups, composed of either two adult females and two juveniles, or four young adults (juveniles from the first groups). Young often co-occur in the same social group as adults but are likely to have a different social role, which may be reflected in their vocal interactions. Therefore, we tested the hypothesis that the social structure of the group influences the parameters of the group vocal network. We found that groups including juveniles presented periods with higher level of activity than groups composed of young adults. Using two types of analyses (Markov analysis and cross-correlation), we showed that juveniles as well as adults were more likely to respond to individuals of their own age-class (i.e. to call one after another, in terms of turn-taking, and within a short time-window, in terms of time delay). When juveniles turned into adulthood, they showed adult characteristics of vocal patterns. Together our results suggest that vocal behavior changes during ontogeny, and individuals are more strongly connected with individuals of the same age-class within acoustic networks.

## Introduction

Social interactions with adults during ontogeny are likely to shape the social developmental trajectories of juvenile individuals. Indeed, some behaviors like courtship, mate choice preferences or foraging skills are partly shaped by social conditions during ontogeny (Freeberg, 1996; Farine et al., 2015) or at adulthood (Freeberg, 2000; Verzijden et al., 2012; Westerman et al., 2012). It has been shown that complex social environments, providing more opportunities for learning, allow individuals to improve their courtship performance or mate choice (during ontogeny, Miller et al., 2008; at adulthood, Oh and Badyaev, 2010; Jordan and Brooks, 2012). For example in brown-headed cowbirds (*Molothrus ater*), young males housed with adult females improvise more song elements than males housed with juvenile females (Miller et al., 2008). Adult females seem to be more selective in their interactions with males than juvenile females, and this study suggests the role of social interactions with adults in young male vocal development (Miller et al., 2008).

Social interactions between peers also take place during ontogeny and may shape the social behavior at adulthood (Bertin et al., 2007; Mariette et al., 2013). For example in zebra finches, the presence of male siblings interferes with the learning of the father's song (Tchernichovski and Nottebohm, 1998). The presence of a female sibling seems to have a positive effect (Adret, 2004). Moreover, it has also been shown that a horizontal transmission of the father's song can occur between two young zebra finch males (Derégnaucourt and Gahr, 2013).

Therefore, studying how juveniles fit into social networks may be central to our understanding of individual developmental trajectories.

Most of the time, social interactions and networks are inferred from proximal measures such as spatial co-occurence or close-contact interaction (Aplin et al., 2013; Farine, 2015; Strandburg-Peshkin et al., 2015). However, it is likely that in groups where members are in close proximity, not all members interact equally with each other, making the social network analysis ineffective in that case. Moreover, in many species, acoustic communication is likely to play a central role in social interactions. However, since acoustic signals can be directed both to individuals at short or long distances, spatial proximity may not necessarily correlate with vocal interactions. Therefore, directly characterizing networks of acoustic communication may be extremely useful for understanding social interactions.

Vocal communication has long been studied in the context of pairwise exchange between one sender and one receiver, but communication networks have progressively received more attention (McGregor, 2005). For example, audience effects are defined as the influence of the presence of other conspecifics on a sender's vocal behavior (Evans and Marler, 1994; Vignal et al., 2004). Eavesdropping is defined as extracting information from signaling interactions while not being the main recipient and seems to occur in many species (McGregor and Dabelsteen, 1996). In birds for example, “eavesdroppers” can respond to vocal exchanges even if they were not part of it initially (Mennill et al., 2002). Multiple individuals may also be involved on both sides of the communication process, such as when a group acts collectively as senders, directing acoustic signals to a group of receivers (Harrington and Mech, 1979; Farabaugh, 1982; Mitani, 1984; McComb et al., 1994).

Vocal communication often relies on temporal and structural regularities in the emission of vocalizations, such as turn-taking (Takahashi et al., 2013; Henry et al., 2015). For example, in humans, turn-taking allows interlocutors to enhance mutual attention and responsiveness (France et al., 2001). Some studies showed that the ability to respect conversation rules, in particular turn-taking may be acquired during development (Hauser, 1992; Miura, 1993; Black and Logan, 1995; Lemasson et al., 2010, 2011; Chow et al., 2015; Takahashi et al., 2016).

The zebra finch (*Taeniopygia guttata*) is a perfectly suited model for studying social interactions during ontogeny using an acoustic communication network. The zebra finch is a socially monogamous and highly social passerine native to the semi-arid zone of Australia, that forages and moves in groups (Zann, 1996). After nutritional independence, juveniles mostly associate with individuals of the same age, with whom some may form affiliative bonds (Zann, 1996). Social experience with peers has developmental consequences, as it affects mating success at adulthood (Mariette et al., 2013). Zebra finches rely heavily on acoustic communication for social interaction (Vignal et al., 2004; Elie et al., 2010; Boucaud et al., 2015; Gill et al., 2015) and start to do so early in life. Indeed, nestlings beg for food and the structure of these begging calls is plastic in response to social interactions with parents (Villain et al., 2015). After fledging, juveniles discriminate the calls of their parents (Jacot et al., 2010; Mulard et al., 2010) and their nest-mates (Ligout et al., 2015) from the calls of other individuals. Young males learn their song by imitation of an adult tutor (Slater et al., 1988). When adult, both males and females utter a repertoire of single-syllable calls while only males sing very stereotyped songs of several syllables (Zann, 1996). Among the calls categories, distance calls are the loudest calls, and convey information on both the sex and the identity of the bird (Vignal et al., 2004, 2008; Forstmeier et al., 2009; Vignal and Mathevon, 2011; Elie and Theunissen, 2016).

The main objective of the present study was to describe zebra finch vocal interactions within an “acoustic network” during ontogeny by comparing the dynamics of vocal interactions of (1) individuals when they were juveniles among adults and (2) the same individuals once they become young adults.

To this aim, we designed a set-up that allows recording of vocal interactions but controls the spatial network. Birds were in individual cages so that they were not able to physically interact and inter-individual distances were fixed. We developed an in-house software that automatically detects vocalizations from hours of passive recording, tags individuals' vocalizations as well as automatically removes non-vocalizations (wings or cage noise) using classification. The resulting vocal signal was analyzed using metrics of vocal activity (number of vocalizations, vocalization rate), vocal timing (cross-correlation), and vocal sequence or turn-taking (Markov analysis).

## Materials and methods

### Subjects and housing conditions

Fifty-six juveniles (28 males and 28 females) aged from 36 to 84 days (mean ± sd: 50.2 ± 10.6, *N* = 56 birds), as well as eight adult females were recorded in the first phase. In the second phase, we recorded the juveniles from phase 1 when they were young adults (48 young adults, including 23 females and 25 males aged from 158 to 230 days). Both phases took place from May 2011 to February 2012. All birds came from our breeding colony (ENES laboratory, University of Saint-Etienne). The juveniles were born in a large indoor aviary (6.5 × 5.5 × 3.5 m; temperature: 20–30°C, daylight: 07:30–20:30) where 28 adult domestic zebra finch pairs were allowed to breed freely and produced 45 broods in total (from April to August 2011). Genetic parents of the broods were not known (because of potential extra-pair copulation and egg dumping), but social parents were known because all juveniles were identified with an individually numbered band before fledging from the nest. After reaching nutritional independence (30–35 days), juveniles were caught in the aviary and transferred to individual cages (40 × 40 × 25) equipped with perches. The eight adult females were also housed in individual cages. In the first phase, adult females were familiar with each other and not with the juveniles, and juveniles were familiar with each other and not with the adult females (juveniles could come from the same nest or not). In the second phase, familiar and unfamiliar young adults (i.e., hold in the same or different rooms between the first and second phases) were present in each group. All birds were kept under the same environmental conditions: temperature between 24 and 26°C, daylight: 07:30–20:30, water, seeds and cuttlefish bones *ad libitum* and supplemented with salad once a week.

### Protocol

**Figure 1:**
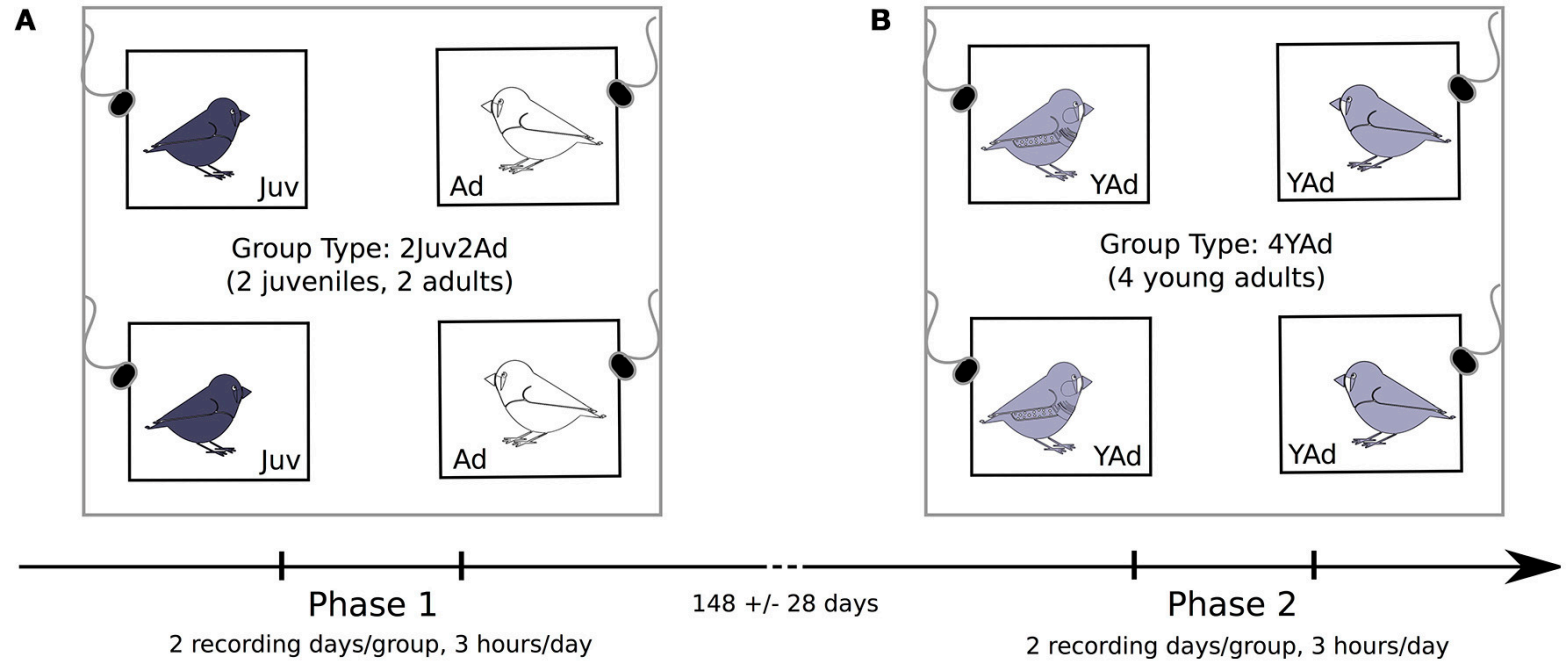
**Schematic of the recording room**. Groups of 4 birds were used, with one bird per cage in each corner of the recording room and one microphone on top of each cage. Two types of groups were tested: **(A)** groups composed of two juveniles and two adult females (Phase 1), and **(B)** groups of four young adults (already tested as juveniles in the first type of group) (Phase 2). The recording duration as well as the average time between the two phases for a given bird is indicated (mean ± sd, *N* = 36).

Recordings took place in a sound-attenuating chamber (2.22 m height × 1.76 m width × 2.28 m length, Silence Box model B, Tip Top Wood, France) fitting four cages (40 × 40 × 25 cm) with one microphone per cage (Figure 1). Cages were separated by 1 m. Microphones (Sennheiser MD42) were connected to a recorder (zoom R16) and suspended from the ceiling 20 cm above the top of the cage. A group of four birds was recorded on two morning sessions, separated by 1 day. On the day between the two sessions, we moved the cages to a second sound-attenuating chamber mimicking the recording chamber. On days of recordings, we moved cages to the recording chamber 15 min before starting the recording. All groups were placed in a sound-attenuating chamber the day before each day of recording so that they could habituate to new surroundings. This protocol allowed studying two groups of four birds in parallel. Each time we moved the cages into a room, we randomly changed the relative positions of the cages so as to control for the potential effect of neighbors' identity and position in the chamber. On each recording day, we recorded vocal exchanges during 3 h starting at 10:30 ± 01:24 (mean ± sd, *N* = 77, recording start time was random according to groups and conditions).

### Groups' composition

We recorded birds during two phases. During the first phase, we recorded groups of four birds made with two adult females and two juveniles of either sex (Figure 1A). During the second phase we recorded groups of four young adults (2 females and 2 males), using the juvenile birds from the first stage (Figure 1B). The time between the two recording phases was on average 148 ± 28 days (mean ± sd, *N* = 36) for a given bird.

### Vocalization extractions

Vocalizations from 250 h of recording were automatically extracted using in-house software. These programs were written in python (http://www.python.org) by authors H.A.S. and M.S.A.F using open-source libraries. This software accuracy was validated and used in previous studies (Elie et al., 2011; Perez et al., 2015). Vocalization detection was a pipeline of three stages.

The first process was a simple threshold-based sound detection based on a high-pass filtered energy envelope (1024 samples FFT; 441 Hz sampling; cut-off frequency: 500 Hz). During the second stage, each sound whose peak was extracted was reconstructed by exploring the two sides of the sound and keeping area with energy higher than 10% of the peak. Thus, each event was either lengthened or shortened to obtain the same amplitude range during the event. This allowed a good estimate of the vocalization duration. The third stage simply merged overlapping waveform segments. Together, the three stages produced start, end, and duration values for each sound event detected in the recording.

Two additional stages enabled to assign each vocalization to its emitter and also remove cage or wing noises. The first additional stage attributed each vocalization to a bird by removing double vocalization, i.e., vocalization emitted by one bird and recorded by its microphone but also recorded by the microphones of all other birds of the group by using energy and delay differences. This allowed us to precisely determine who vocalized at any moment, even in the case of two birds producing two overlapping vocalizations. The second stage removed cage or wings noises using a machine learning process. We trained a supervised classifier using a data set composed of 4500 random extracted sounds from all of our data. Each sound was classified by one expert (MSAF) as “vocalization” or “non-vocalization.” The classification was performed on the spectrogram of the sounds reduced to 50 ms. The idea is to reduce the quantity of information in term of time and frequency, and sample this information in such a way that we will get the same amount of information for each vocalization (short or long). The spectrogram matrix was first reduced to the frequencies of interest—between 500 Hz and 6 kHz. Then two cases appear: if the vocalization duration is longer than 50 ms, we extract 50 ms in the center of the spectrogram, and if the vocalization duration is lower than 50 ms, we keep all the spectrogram and we center it in a 50 ms spectrogram padding the remaining with zeros. The resulting matrix is seen as a vector which contains the flattened spectrogram.

We trained a Random Forest classifier (Breiman, 2001) with 1500 sounds. This classifier had an overall rate of error below 10% of the remaining 3000 sounds.

This procedure allowed us to extract two types of calls from the zebra finch repertoire: tet calls i.e., soft and short harmonic stacks with almost no frequency modulation (Zann, 1975, 1996; Elie and Theunissen, 2016), and distance calls i.e., complex sound consisting of a harmonic series modulated in frequency as well as amplitude (Zann, 1996; Elie and Theunissen, 2016). Males can also perform songs, which are stereotyped series of syllables in a short period of time.

Finally, because we were primarily interested in the temporal dynamic of the exchange, we did not distinguish between different types of vocalizations in the following analyses.

### Data analysis

We separated the analysis in three parts described below: vocal activity, as well as cross-correlations and Markov analyses used to build acoustic networks.

#### Vocal activity

We computed two types of vocal activity metrics. The first type described the group general vocal activity. First we measured the overall vocalization rate, i.e. the total number of vocalizations produced by all individuals in the group divided by the duration of the recording. Then, we measured some characteristics of the vocal bursts. In order to find vocal bursts in a recording, we computed the mean vocalization rate over the whole day, and we extracted the bursts as periods in which the vocalization rate was 10% higher than the mean vocalization rate (with a time step of 1 min with an overlap of 30 s). We then measured the number of bursts, the average vocalization rate in bursts, the burst mean duration, the total duration of bursts in a recording, the inter-burst interval, and the latency to burst (i.e., the time between the recording' start and the beginning of the first burst).

Secondly, we measured the number of vocalizations per individual. We did not need to normalize this number of vocalizations by the recording duration because all recordings lasted the same time (3 h).

#### Cross-correlation

We first characterized the groups' acoustic networks, based on the temporal proximity of vocal activity (functionally equivalent to spatial proximity in co-occurrence networks). In the network, each node is a bird, and the (undirected) edge between two nodes is weighted by the temporal synchrony between the two corresponding birds.

We assessed the vocal temporal synchrony between two birds by computing the cross-correlation using 500 ms time bins. To do that we split the time into 500 ms bins, and each bird signal was one if the bird vocalized within this period, and zero if it did not vocalize. We computed the cross-correlation (cc) between two birds signals with the following formula:

$$\begin{array}{l} \text{cc}=\text{mean}[({\text{S}}_{\text{bird1}}(\text{t})\;-\text{ mean}({\text{S}}_{\text{bird}1}))*({\text{S}}_{\text{bird}2}(\text{t})\\ \;-\text{ mean}({\text{S}}_{\text{bird2}}))]\text{/}(\text{std}(({\text{S}}_{\text{bird}1}))\;*\text{ std}({\text{S}}_{\text{bird}2})) \end{array}$$

Where Sbird1 and Sbird2 are the vocal signals of the two birds as a function of t (time).

The cross-correlation is computed with normalization, i.e., by centering and scaling by the standard deviation (zscoring) of both vocal signals. The result is therefore independent of the total number of vocalizations.

If the cross-correlation shows high positive values, it means that both birds vocalize and remain silent together more often. If the cross-correlation is negative, it means that whenever one bird is vocalizing or silent the other is more often silent or vocalizing respectively.

For each day of recording we computed cross-correlations for all possible dyads of birds.

#### Markov analysis

We then studied the groups' acoustic networks by analyzing the turn-taking.

To establish turn-taking, we only considered the order in which vocalizations were emitted, without consideration of the time between these vocalizations. For that we used Markov chains.

Vocal sequences (taken over the 3 h of recording) were simply transformed into a sequence of caller's identity numbers (e.g. 1,123,113,134). Modeling this as a “four states” process (corresponding to four birds), this vocal sequence can be viewed as a stochastic process that “jumps” from state to state (from one bird to one other). In the Markov hypothesis the caller's identity depends only on the previous caller according to a transition probability (for example the probability of having bird 1 after bird 2). More precisely, a Markov matrix of size 4 × 4 depicts the probability of jumping from one identity to the other: in this matrix, an entry at line i and column j is the probability when the caller is i that the next caller will be j. By construction, this matrix reproduces both the average number of vocalizations for each individual and the first order transition.

We compared the maximum transition probabilities between dyads of birds (e.g., between bird i and bird j, the max transition probability is max(proba(i,j); proba(j,i)), with proba(i,j) the probability for j to vocalize just after i). As for the previous analysis, in the network each node is a bird, and the (undirected) edge between two nodes is weighted by the maximum transition probability between the two corresponding birds.

### Stastistics

All statistical tests were performed using R software (R Core Team, 2014). Linear mixed models were built with the lmer function (lme4 R package), and generalized mixed models were built with the glmer function (lme4 R package) (Bates et al., 2014). Models outputs from “Anova” (car library) (Fox and Weisberg, 2011) and “summary” functions are presented.

#### Model validation

Before being interpreted each model was checked, paying particular attention to its residuals. For generalized linear models with a Poisson family, overdispersion was tested with the “overdisp.glmer” function of the “RVAideMemoire” package (Hervé, 2014), and if the model presented overdispersion we used a negative binomial family. The model validity was also checked with the plotresid function from the “RVAideMemoire” package before interpreting the model results.

#### Model selection

We chose to build biologically relevant models and we kept the full model as recommended by Forstmeier and Schielzeth (2011).

#### Model estimates and confidence intervals

When possible we added information about the quantification of the biological effect given by the models. Confidence intervals were computed with the “confint.merMod” function of the lme4 package. We used the “profile” method for the linear mixed models and the “Wald” method for the negative binomial models.

#### Model random factors

We only kept random factors that had a non-null variance in the model. If we were interested in the significance of the random factors included in the model, we used the following method. We first looked at the values of their residuals in the model summary (“summary” function in lme4 package). We then built two different models: one model including the random factor, and one model without the random factor. We compared these models using the “Anova” function, and if these models were not significantly different we assumed that the random factor effect was not significant. All random factors with non-null variance were kept in the models even if they had no significant effect.

#### Vocal activity

##### Group general vocal activity

**Figure 2:**
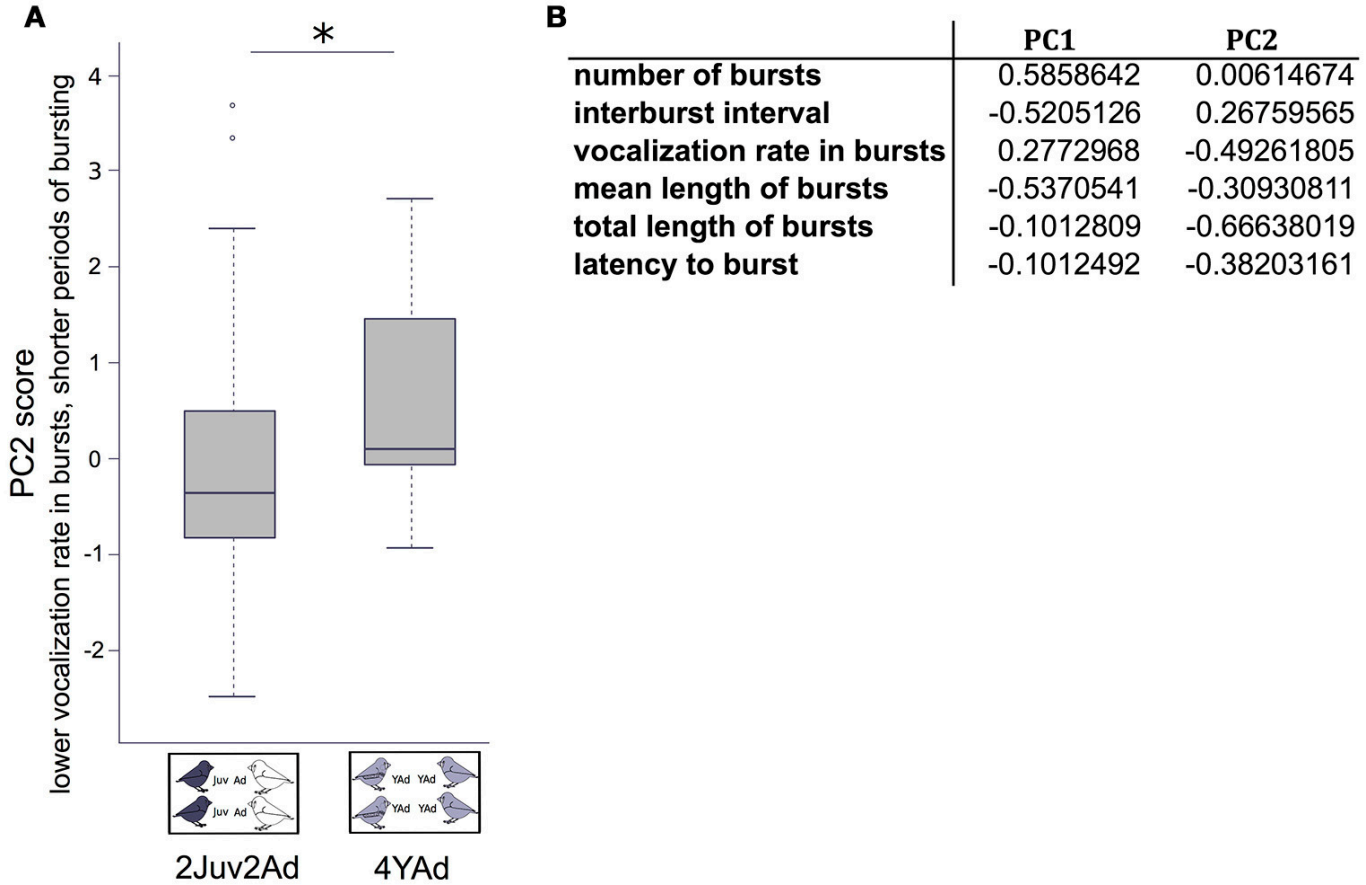
**General vocal activity between group types. (A)** Boxplot of the PC2 values in each group type, from the PCA including six parameters describing the bursts of vocal activity. Linear mixed effect models were built. Detailed sample sizes and model results are given in Table 1. Boxes are median, first and third quartiles (Q1 and Q3 respectively). The upper whisker is located at the ^*^smaller^*^ of the maximum × value and Q3 + 1.5 Inter Quartile Range (IQR), whereas the lower whisker is located at the ^*^larger^*^ of the smallest × value and Q1 − 1.5 IQR. Individual points more extreme in value than Q3 + 1.5 IQR are plotted separately at the high end, and those below Q1 − 1.5 IQR are plotted separately on the low end. **(B)** Variable loadings of the PCA including six parameters on bursts. The first two axes (with eigen-value above 1) explained 88.5% of the data variability. ^*^p < 0.05.

First, for the group general vocal activity we built a Principal Component Analysis (PCA) over six parameters: the number of bursts, the average vocalization rate in bursts, the burst mean duration, the total duration of bursts, the inter-burst interval, and the latency to burst. We found two axes with eigenvalue above 1 that explained 88.5% of the data variability. The first axis describes the general pattern of how bursts were distributed in time (61.7%), and the second axis the density of vocalizations during the recording both within burst and overall (26.8%) (Figure 2).

We built one linear mixed model per PCA axis (PCi) with the following structure:

PCi~GroupType+(1|GroupID)+(1|Day)+(1|StartTime), GroupType having two levels: 2Juv2Ad and 4YAd. The random factors were the group identity (GroupID), the day of recording (Day), and the hour of the recording start (StartTime).

The group type 4YAd had always the same sex ratio (2 females and 2 males). As a second step we restricted the analysis to the first group type 2Juv2Ad alone to study the potential influence of group sex ratio [possible sex ratio for juveniles: 2 males (2M), 2 females (2F) or 1 male and 1 female (1F1M)].

PCi~SexRatio+(1|GroupID)+(1|Day)+(1|StartTime)

##### Number of vocalizations per individual

We built the following generalized mixed linear model (negative binomial family):

$$\begin{array}{l} \text{NVoc}\sim \text{GroupType }*\text{ Sex}+(1|\text{GroupID/BirdID})\;+\;(1|\text{Day})\\ \;+(1|\text{SexRatio})\;+\;(1|\text{StartTime}) \end{array}$$

The response variable was the number of vocalizations. The factor Sex had two levels, M or F. We used a negative binomial model because the model using a Poisson distribution presented overdispersion. The model indicated an interaction between GroupType and Sex at the significance threshold so we studied it using the lsmeans R function.

We built a second model to study the influence of being a juvenile or an adult for GroupType = 2Juv2Ad.

$$\begin{array}{l} \text{NVoc}\sim \text{JuvAd}*\text{SexRatio}+(1|\text{GroupID/BirdID})\\ \;+(1|\text{Day})\;+\;(1|\text{StartTime}) \end{array}$$

The factor JuvAd had two levels: Juv or Ad.

For groups including juveniles, as several factors were linked, we had to build additional models to deal with confounding effects. We built a model using juvenile data only to test the influence of the sex on the number of vocalizations. As the factor SexRatio was strongly linked to the factor Sex we did not include it in this model:

$$\begin{array}{l} \text{NVocJuveniles}\sim \text{Sex}+(1|\text{GroupID/BirdID})\;+\;(1|\text{Day})\\ \;+(1|\text{StartTime}) \end{array}$$

We then built a model using the females' data only to test the difference between adult and juvenile females (as the males were juveniles only).

$$\begin{array}{l} \text{NVocFemales}\sim \text{JuvAd}+(1|\text{Group/BirdID})\;+\;(1|\text{Day})\\ \;+(1|\text{StartTime}) \end{array}$$

#### Cross correlation

First we built a model in order to compare the cross-correlation between group types (2Juv2Ad and 4YAd):

$$\begin{array}{l} \text{cc}\sim \text{GroupType}*\text{Sex}1\text{Sex}2\;+\text{ Dist }+\text{ Dist}:\text{GroupType}\\ +\text{Dist}:\text{Sex}1\text{Sex}2\;+\;(1|\text{GroupID})\;+\;(1|\text{Day})\;+\;(1|\text{Bird1ID})\\ +(1|\text{Bird2ID})\;+\;(1|\text{StartTime}) \end{array}$$

The distance between two birds could be 1 or 2 (1: birds were on the same edge of the square, 2: birds were placed on the diagonal). The factor Sex1Sex2 had three levels: FF, MM, or FM and represented the sexes of both birds from which we computed the cross-correlation.

As the interaction between the group type and the sex was significant we first separated the dataset by group type and analyzed them separately:

$$\begin{array}{l} \text{GroupType}=\text{4YAd}:\\ \text{cc}\sim \text{Sex1Sex2 }*\text{ Dist }+\;(1|\text{GroupID})\\ +\;(1|\text{Day})\;+\;(1|\text{Bird1ID})\;+\;(1|\text{Bird2ID})+\;(1|\text{StartTime}) \end{array}$$

GroupType = 2Juv2Ad:

the factor Sex1Sex2 was strongly linked to the factors JuvAd (three levels: JuvJuv, AdAd, JuvAd) which indicated if the dyads of birds comprised only juveniles, only adults or one juvenile and one adult and SexRatio (as the SexRatio could differ between groups), therefore we first built the following model including factors SexRatio and JuvAd: cc~JuvAd+Dist+SexRatio+JuvAd:Dist+JuvAd:SexRatio +(1|GroupID)+(1|Day)+(1|Bird1ID)+(1|Bird2ID)+(1|StartTime)

We then separated the dataset by sexes to assess the difference between the cross-correlations of two juveniles and two young adults. As we had only one data point per bird in this case, the only remaining random factor is Day. For each value of Sex1Sex2 (MM, MF, FF) we built the following model:

cc~GroupType + (1|Day) + (1|StartTime)

#### Markov analysis

We first built a model to compare the maximum transition probabilities between group types (2Juv2Ad and 4YAd):

$$\begin{array}{l} \text{MaxProba}\sim \text{GroupType }*\text{ Sex1Sex2 }+\text{ Dist}\\ \;+\text{ Dist}:\text{GroupType }+\text{ Dist}:\text{Sex1Sex2}\\ \;+\;(1|\text{GroupID})+\;(1|\text{Day})\;+\;(1|\text{Bird1ID})\\ \;+\;(1|\text{Bird2ID})\;+\;(1|\text{StartTime}) \end{array}$$

As the interaction between GroupType and Sex1Sex2 was significant we analyzed the group types separately, as we did for the cross-correlation.

$$\begin{array}{l} \text{Juveniles only}:\text{ MaxProba}\sim \text{Sex1Sex2 }*\text{ Dist }+\;(1|\text{GroupID})\\ \;+\;(1|\text{Day})\;+\;(1|\text{Bird1ID})\\ \;+\;(1|\text{Bird2ID})\;+\;(1|\text{StartTime}) \end{array}$$

## Results

### Vocal activity

#### Group general vocal activity

We found an effect of the group type on the second composite score of the PCA, which mainly depicted the vocalization rate in bursts and the total length of bursts. Groups including juveniles and adults presented lower scores in PC2 than groups including only young adults, which means that vocalization rate in bursts and total duration of bursting was higher in the former than in the latter (Figure 2, Table 1). We found no effect of the group type or sex ratio on the first composite score of the PCA (number of bursts, inter-burst interval, mean length of bursts) (Table 1).

**Table 1:** **Impact of group type and sex ratio on general vocal activity**.

**PC2~GroupType+(1|GroupID)+(1|Day)**					
**Nobs = 70, N2Juv2Ad = 52, N4YAd = 18**					
*Random Effects*					
**Groups name**	**Variance**	**Std. Dev**.			
GroupID (intercept)	0.1074	0.3277			
Day (intercept)	2.347e-16	1.532e-08			
Residual	1.429	1.196			
*Fixed Effects*					
	**Estimate**	**Std. Error**	**df**	***t*****-value**	***p*****-value**
(Intercept)	−0.1823	0.1757	34.9	−1.037	0.3066
GroupType-4YAd	0.7394	0.3545	41.35	2.086	0.0432
**PC2~SexRatio+(1|StartTime)**					
**Nobs = 52, N2F2M = 15, N3F1M = 23, N4F0M = 14**					
*Random Effects*					
**Groups name**	**Variance**	**Std. Dev**.			
StartTime(intercept)	0.119	0.245			
Residual	1.478	1.216			
*Fixed Effects*					
	**Estimate**	**Std. Error**	**df**	***t*****-value**	***p*****-value**
(Intercept)	−0.5437	0.3859	6.010	−1.409	0.208
SexRatio-3F1M	0.3923	0.4067	47.93	2.086	0.340
SexRatio-4F0M	0.7170	0.4634	48.99	1.547	0.128

*Model statistical results are shown. Linear mixed effect models (“lmer” function from “lme4” R package) were built. Number of observations in the dataset for each fixed effect is given. We present the results from the R “summary” function*.

#### Number of vocalizations per individual

**Figure 3:**
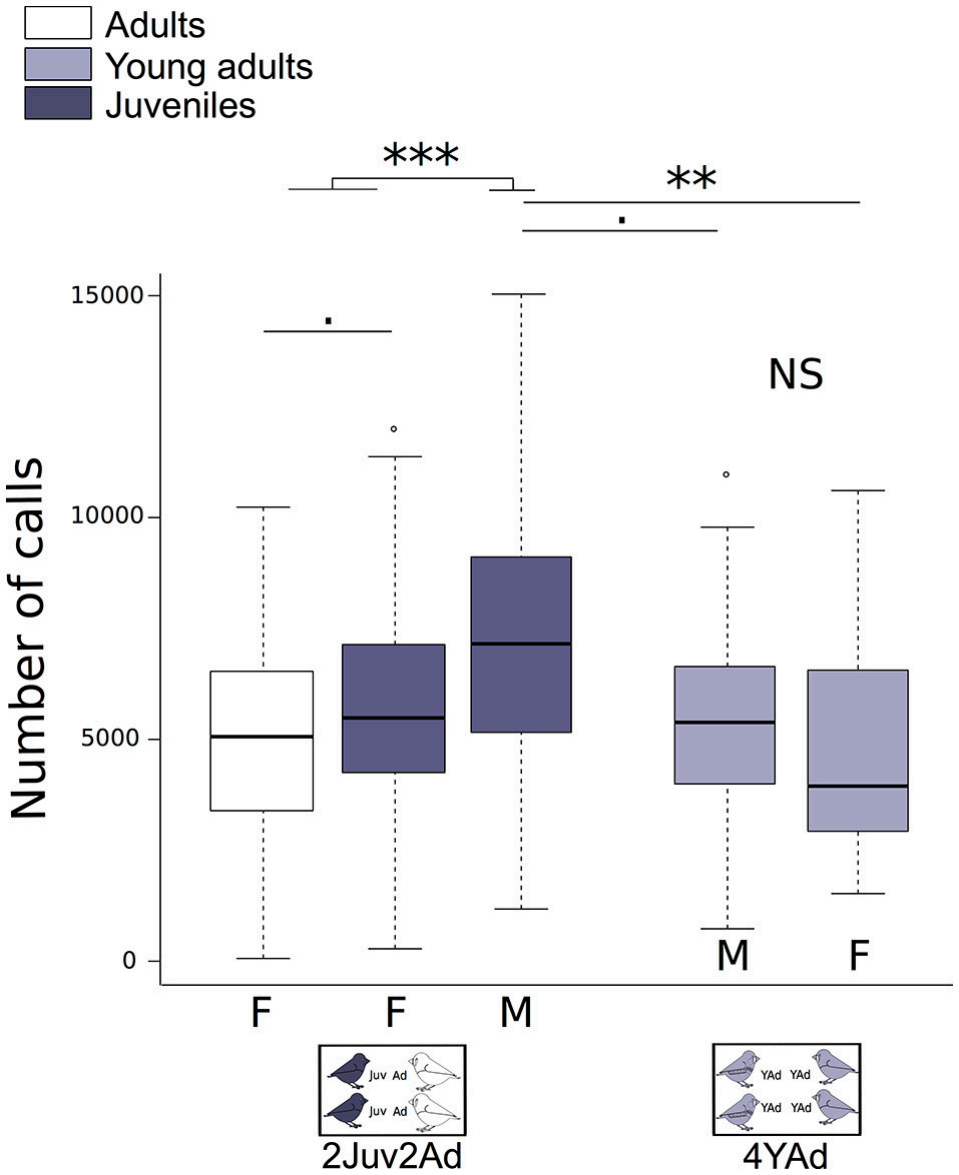
**Number of vocalizations per individual**. Boxplot of individual vocalization rates in the different group types. Number of vocalizations per individual for each sex is detailed, as well as the age category (Juveniles, Young Adults, Adults). Generalized linear mixed effect models with negative binomial family were built. Detailed sample sizes and model results are given in Table 2. Boxes are median, first and third quartiles (Q1 and Q3 respectively). The upper whisker is located at the ^*^smaller^*^ of the maximum × value and Q3 + 1.5 Inter Quartile Range (IQR), whereas the lower whisker is located at the ^*^larger^*^ of the smallest × value and Q1 − 1.5 IQR. Individual points more extreme in value than Q3 + 1.5 IQR are plotted separately at the high end, and those below Q1 − 1.5 IQR are plotted separately on the low end. ^***^p < 0.001, ^**^p < 0.1, *p* < 0.1.

We found differences between group types depending on the sex (Figure 3). The juvenile males emitted more vocalizations than all other birds (adults, young adults, and juvenile females). Adults emitted less vocalizations than juveniles. This difference was more pronounced for juvenile males than juvenile females (Figure 3, Table 2). Vocalization rate in juveniles was 1.34 times [1.03;1.71] higher than in adults (numbers in brackets are 95% confidence interval of the effect estimated by the model). Among juveniles, the vocalization rate was 1.39 times [1.18;1.63] higher in males than in females. Male songs may increase the number of vocalizations. To account for the song occurrence, we counted the total number of detected song syllables (from all males) over 10 min (randomly chosen from 1 day) for each group (i.e., we counted songs over 3.5 h of recording in total), which we compared to the total number of detected vocalizations of these males. For juveniles we found that song syllables represented only 2.3 ± 7% of the total detected vocalizations in males. Individual changes in vocalization rate along ontogeny are shown in Supplementary Figure 1 (females) and Supplementary Figure 2 (males).

**Table 2:** **Impact of group composition on the number of vocalizations per individual**.

NVoc~GroupType^*^Sex+(1|GroupID/BirdID)+(1|Day) +(1|SexRatio)					
**Nobs = 319, N2Juv2Ad = 227, N4YAd = 92, Nmale = 103, Nfemale = 216**					
*Random Effects*					
**Groups name**	**Variance**	**Std. Dev**.			
GroupID/BirdID (intercept)	8.823e-15	9.393e-08			
GroupID (intercept)	1.200e-02	1.095e-01			
Day (intercept)	5.046e-04	2.246e-02			
SexRatio (intercept)	3.110e-14	1.764e-07			
Residual	7.534e-01	8.680e-01			
*Fixed Effects*					
	**Estimate**	**Std. Error**	***t*****-value**	***p*****-value**	
(Intercept)	8.569	0.0504	169.9	<0.0001	
GroupType-4YAd	−0.1549	0.0997	−1.55	0.1202	
Sex-M	0.3012	0.0819	3.68	0.0002	
GroupType-4YAd: Sex-M	−0.1441	0.1376	−1.05	0.2946	
*Pairwise Comparisons (Tukey Adjustment)*					
**Contrast**	**Estimate**	**SE**	**df**	***z*****-ratio**	***p*****-value**
F2Juv2Ad-M2Juv2Ad	−0.3230	0.0818	NA	−3.944	0.0005
F2Juv2Ad-F4YAd	0.0844	0.0973	NA	0.8671	0.8219
F2Juv2Ad-M4YAd	−0.0471	0.0936	NA	−0.5039	0.9582
M2Juv2Ad-F4YAd	0.4074	0.1143	NA	3.561	0.0021
M2Juv2Ad-M4YAd	0.2758	0.1112	NA	2.480	0.063
F4YAd-M4YAd	−0.1316	0.1193	NA	−1.102	0.688
NVoc~JuvAd ^*^ SexRatio + (1| GroupID/BirdID) +(1|Day)+(1|StartTime)					
**Nobs = 227, NAd = 116, NJuv = 111, N2F2M = 64, N3F1M = 92, N4F0M = 56**					
*Random Effects*					
**Groups name**	**Variance**	**Std. Dev**.			
GroupID/BirdID (intercept)	2.567e-14	1.602e-07			
GroupID (intercept)	2.159e-09	4.647e-05			
Day (intercept)	7.536e-19	8.681e-10			
StartTime (intercept)	2.186e-14	1.478e-07			
Residual	6.524e-01	8.077e-01			
*Fixed Effects*					
	**Estimate**	**Std. Error**	***t*****-value**	***p*****-value**	
(Intercept)	8.6395	0.0964	89.62	<0.0001	
JuvAd-Juv	0.3087	0.1363	2.26	0.0236	
SexRatio-3F1M	−0.1258	0.1239	−1.02	0.3100	
SexRatio-4F0M	−0.1997	0.1387	−1.44	0.1501	
JuvAd-Juv: SexRatio-3F1M	−0.0332	0.1752	−0.19	0.8496	
JuvAd-Juv: SexRatio-4F0M	−0.2060	0.1962	−1.05	0.2938	
**NVocJuveniles~Sex +(1|GroupID/BirdID)**					
**Nmale = 55, Nfemale = 56**					
*Random Effects*					
**Groups name**	**Variance**	**Std. Dev**.			
GroupID/BirdID (intercept)	0.0	0.0			
GroupID (intercept)	0.0113	0.1067			
Residual	0.7419	0.8614			
*Fixed Effects*					
	**Estimate**	**Std. Error**	***t*****-value**	***p*****-value**	
(Intercept)	8.65277	0.06956	125.39	<0.0001	
Sex-M	0.21906	0.09865	2.22	0.0264	
**NVocFemales~JuvAd +(1|GroupID)**					
**NAd = 116, NJuv = 56**					
*Random Effects*					
**Groups name**	**Variance**	**Std. Dev**.			
GroupID (intercept)	0.0029	0.0542			
Residual	0.6510	0.8068			
*Fixed Effects*					
	**Estimate**	**Std. Error**	***t*****-value**	***p*****-value**	
(Intercept)	8.4999	0.05545	153.3	<0.0001	
JuvAd-Juv	0.1452	0.0931	1.56	0.119	

*Model statistical results are shown. Generalized linear mixed effect models with negative binomial family (“lmer” function from “lme4” R package) were built. Number of observations in the dataset for each fixed effect is given. We present the results from the R “summary” function*.

### Cross correlation

**Figure 4:**
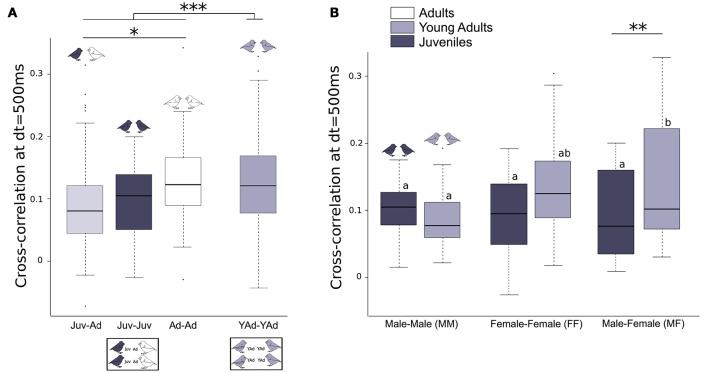
**Vocal cross-correlation between two birds**. Boxplot of cross-correlation values at dt = 500 ms, between two birds **(A)** of different age categories (Juv-Juv, Juv-Ad, Ad-Ad, YAd-Yad), **(B)** of different sex (MM, MF, FF), within a given age category (Juv or YAd). Linear mixed effect models were built. Detailed sample sizes and model results are given in Table 3. Different letters indicate significant differences. Boxes are median, first and third quartiles (Q1 and Q3 respectively). The upper whisker is located at the ^*^smaller^*^ of the maximum × value and Q3 + 1.5 Inter Quartile Range (IQR), whereas the lower whisker is located at the ^*^larger^*^ of the smallest × value and Q1 − 1.5 IQR. Individual points more extreme in value than Q3 + 1.5 IQR are plotted separately at the high end, and those below Q1 − 1.5 IQR are plotted separately on the low end. ^***^p < 0.001, ^**^p < 0.1, ^*^p < 0.05, *p* < 0.1.

Young adult groups presented significantly higher cross-correlation values than groups of juveniles and adults. We found that cross-correlation values (i.e., temporal synchrony of vocalizations) between one juvenile and one adult (Juv-Ad) were lower than those between two adults (Ad-Ad). Cross-correlation values between two juveniles (Juv-Juv) were intermediate (Figure 4A, Table 3). Supplementary Figure 3 illustrates these results with four examples of groups with juveniles.

**Table 3:** **Impact of group composition on the vocal cross-correlation**.

CrossCorr~GroupType^*^Sex1Sex2+Dist+Dist:GroupType+Dist:Sex1Sex2+(1|GroupID)+(1|Bird1ID)+(1|Bird2ID)+(1|Day)+(1|StartTime)						
**Nobs = 486, N2Juv2Ad = 348, N4YAd = 138, NFF = 205, NMF = 223, NMM = 43, NDist1 = 405, NDist2 = 81**						
*Random Effects*						
**Groups name**	**Variance**		**Std. Dev**.			
GroupID (intercept)	1.084e-03		0.0329			
Bird1ID (intercept)	2.285e-04		0.0151			
Bird2ID (intercept)	1.695e-04		0.0130			
Day (intercept)	2.507e-05		0.0050			
StartTime (intercept)	8.521e-05		0.0092			
Residual	2.378e-03		0.0487			
*Fixed Effects*						
		**Estimate**	**Std. Error**	**df**	***t*****-value**	***p*****-value**
(Intercept)		0.0906	0.0107	12.9	8.440	<0.0001
GroupType-4YAd		0.0570	0.0180	93	3.156	0.0021
Sex-MF		−0.0152	0.0079	300.9	−1.902	0.0581
Sex-MM		0.0067	0.0177	282.6	0.382	0.7024
Dist-2		0.0252	0.0099	375.2	2.530	0.0118
GroupType-4YAd: Sex-MF		−0.0052	0.0148	417.8	−0.354	0.7233
GroupType-4YAd: Sex-MM		−0.0701	0.0228	394.6	−3.072	0.0022
GroupType-4YAd: Dist-2		−0.0260	0.0168	386.1	−1.549	0.1222
Sex-MF: Dist-2		0.0221	0.0159	388	1.391	0.1650
Sex-MM: Dist-2		0.0316	0.0271	363.9	1.164	0.2453
CrossCorr4YAd~Sex1Sex2^*^Dist+(1|GroupID)+(1|Bird1ID)+(1|Bird2ID) +(1|Day)						
**Nobs = 138, NFF = 21, NMF = 90, NMM = 27, NDist1 = 115, NDist2 = 23**						
*Random Effects*						
**Groups name**		**Variance**	**Std. Dev**.			
GroupID (intercept)		0.0032	0.0572			
Bird1ID (intercept)		0.0006	0.0258			
Bird2ID (intercept)		0.0004	0.0208			
Day (intercept)		0.0005	0.0240			
StartTime (intercept)		0.0007	0.0267			
Residual		0.0017	0.0416			
*Fixed Effects*						
		**Estimate**	**Std. Error**	**df**	***t*****-value**	***p*****-value**
(Intercept)		0.1422	0.0330	4.03	4.304	0.0124
Sex-MF		−0.0204	0.0124	103.83	−1.649	0.1021
Sex-MM		−0.0639	0.0177	60.19	−3.601	0.0006
Dist-2		0.0589	0.0425	29.4	1.384	0.1767
Sex-MF: Dist-2		−0.0422	0.0450	34.51	−0.937	0.3553
*Pairwise Comparisons (Tukey Adjustment)*						
Contrast		**Estimate**	**SE**	***z*****-value**	***p*****-value**	
Sex-MF-Sex-FF = = 0		−0.0204	0.0124	−1.649	0.2117	
Sex-MM-Sex-FF = = 0		−0.0639	0.0177	−3.601	<0.0001	
Sex-MM-Sex-MF = = 0		−0.0434	0.0122	−3.560	0.0011	
**CrossCorr2Juv2Ad~JuvAd+Dist+SexRatio+JuvAd:Dist+JuvAd:SexRatio+(1|GroupID)+ (1|Bird1ID)+(1|Bird2ID)+(1|StartTime)**						
**Nobs = 348, NAdAd = 58, NJuvAd = 232, NJuvJuv = 58, NDist1 = 290, NDist2 = 58, N2F2M = 96, N3F1M = 138, N4F0M = 84**						
*Random Effects*						
**Groups name**		**Variance**	**Std. Dev**.			
GroupID (intercept)		6.924e-04	0.0263			
Bird1ID (intercept)		8.076e-05	0.0089			
Bird2ID (intercept)		1.685e-04	0.0129			
StartTime (intercept)		8.826e-05	0.0093			
Residual		2.304e-03	0.0480			
*Fixed Effects*						
		**Estimate**	**Std. Error**	**df**	***t*****-value**	***p*****-value**
(Intercept)		1.193e-01	1.814e-02	52.77	6.577	<0.0001
JuvAd-JuvAd		−3.949e-02	1.507e-02	271.2	−2.620	0.0092
JuvAd-JuvJuv		−1.844e-02	2.004e-02	150.8	−0.920	0.3590
Dist-2		8.799e-03	2.123e-02	270.8	0.414	0.6788
SexRatio-3F1M		2.976e-02	2.035e-02	98.93	1.463	0.1467
SexRatio-4F0M		−2.933e-03	2.312e-02	91.45	−0.127	0.8993
JuvAd-JuvAd: Dist-2		3.538e-02	2.348e-02	273.4	1.507	0.1330
JuvAd-JuvJuv: Dist-2		3.147e-02	2.762e-02	278.4	1.139	0.2555
JuvAd-JuvAd: SexRatio-3F1M		−2.190e-02	1.844e-02	260.1	−1.188	0.2360
JuvAd-JuvJuv: SexRatio-3F1M		−4.473e-02	2.375e-02	247.4	−1.883	0.0608
JuvAd-JuvAd: SexRatio-4F0M		4.872e-04	2.052e-02	257.9	0.024	0.9810
JuvAd-JuvJuv: SexRatio-4F0M		−1.620e-02	2.651e-02	259.0	−0.611	0.5417
*Pairwise Comparisons (Tukey Adjustment)*						
**Contrast**		**Estimate**	**SE**	***z*****-value**	***p*****-value**	
JuvAd-AdAd = = 0		−0.0394	0.0150	−2.620	0.0229	
JuvJuv-AdAd = = 0		−0.0184	0.0200	−0.920	0.6197	
JuvJuv-JuvAd = = 0		0.0210	0.0152	1.385	0.3405	
**CrossCorrMM~GroupType+(1|Day)**						
**Nobs = 48, N2Juv2Ad = 16, N4YAd = 27**						
*Random Effects*						
**Groups name**		**Variance**	**Std. Dev**.			
Day (intercept)		0.00	0.00			
Residual		0.0022	0.0470			
*Fixed Effects*						
		**Estimate**	**Std. Error**	**df**	***t*****-value**	***p*****-value**
(Intercept)		0.1009	0.0117	37	8.58	<0.0001
GroupType-4YAd		−0.0148	0.0153	37	−0.967	0.34
*Fixed Effects*						
		**Estimate**	**Std. Error**	**df**	***t*****-value**	***p*****-value**
(Intercept)		0.0939	0.0130	95	7.196	<0.0001
GroupType-4YAd		0.0409	0.0149	95	2.740	0.0073
**CrossCorrFF~GroupType+(1|Day)**						
**Nobs = 40, N2Juv2Ad = 14, N4YAd = 21**						
*Random Effects*						
**Groups name**		**Variance**	**Std. Dev**.			
Day (intercept)		0.00	0.00			
Residual		0.0073	0.0856			
*Fixed Effects*						
		**Estimate**	**Std. Error**	**df**	***t*****-value**	***p*****-value**
(Intercept)		0.0917	0.0229	29	4.005	3.95e-04
GroupType-4YAd		0.0586	0.0309	29	1.895	0.0680

*Model statistical results are shown. Linear mixed effect models (“lmer” function from “lme4” R package) were built. Number of observations in the dataset for each fixed effect is given. We present the results from the R “summary” function*.

We also found sex differences between groups: synchrony between 1 male and 1 female increased from juveniles to young adults, whereas it remained the same between 2 males or 2 females (Figure 4B, Table 3). Specifically, female-male dyads increased their cross-correlation value from 0.09 [0.07;0.12] (juveniles) to 0.13 [0.10;0.16] (young adults). There was no cross-correlation difference between the sexes within groups including juveniles and adults. Also, there was no difference in cross-correlation between the 2 days of recording.

### Markov analysis

**Figure 5:**
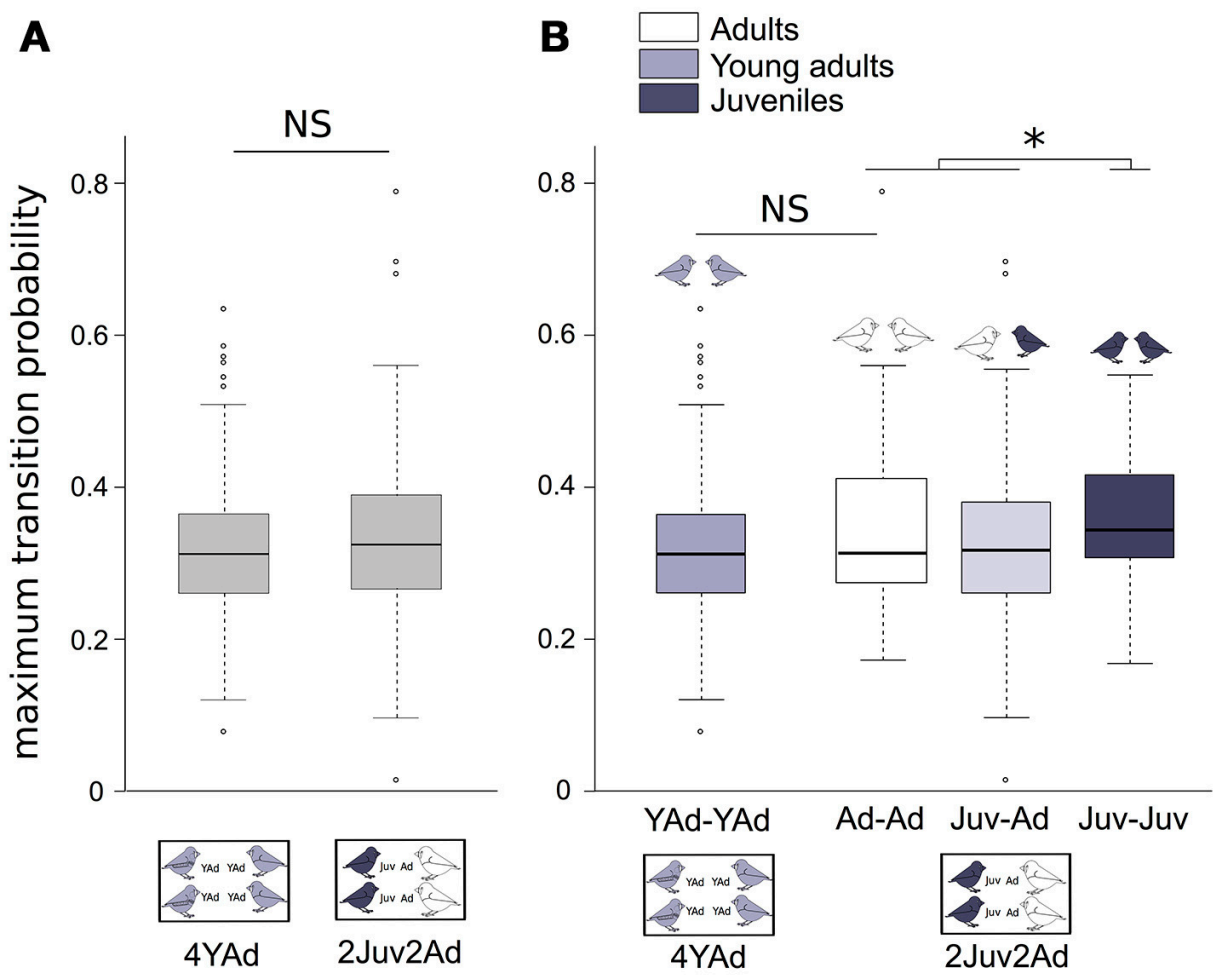
**Mean vocal transition probabilities between two birds**. Boxplot of mean transition probabilities between two birds **(A)** in the different group types, **(B)** from different age categories (Juv-Juv, Juv-Ad, Ad-Ad, YAd-Yad). Linear mixed effect models were built. Detailed sample sizes and model results are given in Table 4. Boxes are median, first and third quartiles (Q1 and Q3 respectively). The upper whisker is located at the ^*^smaller^*^ of the maximum × value and Q3 + 1.5 Inter Quartile Range (IQR), whereas the lower whisker is located at the ^*^larger^*^ of the smallest × value and Q1 − 1.5 IQR. Individual points more extreme in value than Q3 + 1.5 IQR are plotted separately at the high end, and those below Q1 − 1.5 IQR are plotted separately on the low end. ^*^p < 0.05.

The maximum transition probabilities (i.e., turn-taking) did not differ between group types (Figure 5A, Table 4).

**Table 4:** **Impact of group composition on the dyads average mean transition probabilities**.

MaxProba~GroupType ^*^ Sex1Sex2+Dist+ Dist:GroupType+ Dist:Sex1Sex2+ (1|GroupID)+ (1|Bird1ID)+ (1|Bird2ID)+ (1|Day)						
**Nobs = 457, N2Juv2Ad = 344, N4YAd = 134, NFF = 197, NMF = 206, NMM = 39, NDist1 = 380, NDist2 = 77**						
*Random Effects*						
**Groups name**	**Variance**	**Std. Dev**.				
GroupID (intercept)	0.0112	0.1059				
Bird1ID (intercept)	0.0093	0.0968				
Bird2ID (intercept)	6.197e-04	0.0248				
Day (intercept)	1.068e-04	0.0103				
Residual	0.0129	0.1139				
*Fixed Effects*						
	**Estimate**	**Std. Error**	**df**	***t*****-value**	***p*****-value**	
(Intercept)	−0.8655	0.0324	53.4	−26.693	<0.0001	
GroupType-4YAd	0.0453	0.0443	190.3	1.024	0.3073	
Sex-MF	0.0471	0.0357	62.7	1.317	0.1925	
Sex-MM	0.1208	0.0595	144	2.029	0.0443	
Dist-2	0.0079	0.0234	294.1	0.338	0.7357	
GroupType-4YAd: Sex-MF	−0.0690	0.0482	355.6	−1.433	0.1528	
GroupType-4YAd: Sex-MM	−0.2073	0.0669	418.2	−3.095	0.0021	
Sex-MF: Dist-2	0.0122	0.0380	294.1	0.323	0.7467	
Sex-MM: Dist-2	−0.0398	0.0668	286.8	−0.597	0.551	
GroupType-4YAd: Dist-2	−0.0222	0.0431	279	−0.516	0.6064	
MaxProba4YAd~ Sex1Sex2^*^Dist+ (1|GroupID)+ (1|Bird1ID)+ (1|Bird2ID)+ (1|Day)						
**Nobs = 114, NFF = 17, NMF = 74, NMM = 23, NDist1:95, NDist2 = 19**						
*Random Effects*						
**Groups name**	**Variance**	**Std. Dev**.				
GroupID (intercept)	6.032e-17	7.767e-09				
Bird1ID (intercept)	9.097e-03	9.538e-02				
Bird2ID (intercept)	1.371e-02	1.171e-01				
Day (intercept)	2.129e-04	1.459e-02				
StartTime (intercept)	4.058e-05	6.370e-03				
Residual	1.528e-02	1.236e-01				
*Fixed Effects*						
	**Estimate**	**Std. Error**	**df**	***t*****-value**	***p*****-value**	
(Intercept)	−0.8186	0.0537	29.28	−15.221	<0.0001	
Sex-MF	−0.0447	0.0567	40.71	−0.789	0.4349	
Sex-MM	−0.1229	0.0703	50.05	−1.749	0.0864	
Dist-2	−0.0040	0.1522	66.17	−0.027	0.9788	
Sex-MF: Dist-2	−0.0117	0.1581	65.38	−0.074	0.9410	
*Pairwise Comparisons (Tukey Adjustment)*						
**Contrast**	**Estimate**	**SE**	***z*****-value**	***p*****-value**		
Sex-MF-Sex-FF = = 0	−0.0447	0.0567	−0.789	0.703		
Sex-MM-Sex-FF = = 0	−0.1229	0.0703	−1.749	0.181		
Sex-MM-Sex-MF = = 0	−0.0781	0.0488	−1.601	0.238		
**MaxProba2Juv2Ad~JuvAd+ Dist+ SexRatio+JuvAd:Dist+ JuvAd:SexRatio+ (1|GroupID)+ (1|Bird1ID)+(1|Bird2ID)**					
**Nobs = 344, NAdAd = 57, NJuvAd = 229, NJuvJuv = 58, NDist1:286, NDist2 = 58, N2F2M = 96, N3F1M = 138, N4F0M = 80**						
*Random Effects*						
**Groups name**	**Variance**	**Std. Dev**.				
GroupID (intercept)	0.0023	0.0489				
Bird1ID (intercept)	0.0068	0.0829				
Bird2ID (intercept)	0.0102	0.1012				
Residual	0.0085	0.0924				
*Fixed Effects*						
	**Estimate**	**Std. Error**	**df**	***t*****-value**	***p*****-value**	
(Intercept)	−0.8494	0.0575	47.29	−14.754	<0.0001	
JuvAd-JuvAd	0.0065	0.0462	131.61	0.141	0.8881	
JuvAd-JuvJuv	0.1351	0.0711	57.81	1.900	0.0625	
Dist-2	0.0075	0.0424	233.88	0.178	0.8589	
SexRatio-3F1M	0.0569	0.0402	55.95	1.414	0.1628	
SexRatio-4F0M	0.0963	0.0456	52.21	2.112	0.0395	
JuvAd-JuvAd: Dist-2	0.0229	0.0476	234.04	0.481	0.6311	
JuvAd-JuvJuv: Dist-2	−0.0215	0.0551	216.05	−0.392	0.6957	
JuvAd-JuvAd: SexRatio-3F1M	−0.0282	0.0452	237.88	−0.624	0.5335	
JuvAd-JuvJuv: SexRatio-3F1M	−0.1225	0.0671	122.41	−1.825	0.0704	
JuvAd-JuvAd: SexRatio-4F0M	−0.0707	0.0512	259.63	−1.381	0.1683	
JuvAd-JuvJuv: SexRatio-4F0M	−0.1498	0.0761	143.71	−1.969	0.0509	
*Pairwise Comparisons (Tukey Adjustment)*						
**Contrast**	**Estimate**	**SE**	***z*****-value**	***p*****-value**		
JuvAd-AdAd = = 0	0.0065	0.0462	0.141	0.9879		
JuvJuv-AdAd = = 0	0.1351	0.0711	1.900	0.1243		
JuvJuv-JuvAd = = 0	0.1286	0.0460	2.792	0.0129		
MaxProbaJuvJuv~Sex1Sex2^*^Dist+(1|Bird1ID)+(1|Day)						
**Nobs = 58, NFF = 14, NMF = 23, NMM = 16, NDist1 = 47, NDist2 = 11**						
*Random Effects*						
**Groups name**	**Variance**	**Std. Dev**.				
Bird1ID (intercept)	0.0053	0.0733				
Day (intercept)	0.0023	0.0480				
Residual	0.0133	0.1155				
*Fixed Effects*						
	**Estimate**	**Std. Error**	**df**	***t*****-value**	***p*****-value**	
(Intercept)	−0.8258	0.0559	3.42	−14.754	0.0003	
Sex-MF	0.0398	0.0568	25.26	0.701	0.4894	
Sex-MM	0.1006	0.0618	28.05	1.628	0.1147	
Dist-2	−0.0442	0.0825	33.85	−0.536	0.5955	
Sex-MF: Dist-2	0.0349	0.1070	32.44	0.326	0.7461	
Sex-MM: Dist-2	0.0091	0.1100	33.95	0.083	0.9341	

*Model statistical results are shown. Linear mixed effect models (“lmer” function from “lme4” R package) were built. Number of observations in the dataset for each fixed effect is given. We present the results from the R “summary” function*.

The maximum transition probabilities were higher between two juveniles than between other dyads (AdAd, two adults or JuvAd, one adult and one juvenile). Thus, juveniles were more likely to vocalize after another juvenile's vocalization in the turn-taking sequence. The average of maximum transition probability was the same between two adults or two young adults (Figure 5B, Table 4). Also, there was no difference in transition probabilities between the 2 days of recording.

## Discussion

Using our in-house software we were able to automatically detect vocalizations from hours of passive recordings in groups of four zebra finches. This allowed us to assess information about the acoustic network of groups composed of adults and juveniles compared to groups of only young adults. We found that groups including juveniles presented periods with higher level of activity than groups composed of young adults only and within their groups, juveniles vocalized more than adults. Furthermore, we saw that two adults were more likely to vocalize together within a short time window (cross-correlation) than one adult and one juvenile, and that juveniles were more likely to vocalize after one another in turn-taking sequences (Markov analysis). Finally, when juveniles turned into adulthood, they showed adult characteristics of vocal patterns (number of vocalizations, cross-correlation, turn-taking).

Groups including juveniles had a higher vocalization rate during bursts, and these bursts lasted longer. At the individual level, juveniles had a higher vocalization rate than adults or young adults. First, juveniles could be more active in general in their behavior than adults. Indeed, in several species the locomotor activity is higher in young individuals than in older individuals (Van Waas and Soffié, 1996; Ingram, 2000). By vocalizing more, juveniles get opportunities to vocally interact in a greater diversity of contexts, which may be important to develop their social skills. In cowbirds, it has been shown that a complex social environment (in which birds changed regularly of social groups) can lead to a greater social competence and also a higher mating success (White et al., 2010). Vocalizing more might also allow juveniles to practice conversation rules, and more precisely to learn to respect turn-taking rules. Indeed, some studies show that the ability to respect turns may be acquired during development (Hauser, 1992; Miura, 1993; Black and Logan, 1995; Lemasson et al., 2010, 2011; Chow et al., 2015; Takahashi et al., 2016).

Juvenile males' vocalization rate was higher than juvenile females' vocalization rate. Two potential interpretations need to be addressed here. First, this result could be due to our method, which is not able to discriminate between calls and songs' syllables. However, as indicated in the results, we concluded that the contribution of songs represented an average of 2.3% of all male vocalizations. This could not account for the difference between juvenile males and females' number of vocalizations, because males gave 24.8% more vocalizations than females. Second, the two adults with the juveniles were always two adult females. Juvenile males may vocalize more than juvenile females in the presence of adult females (and not adult males). A previous study analyzed the response of zebra finch juveniles (aged of 56.5 ± 2.4 days) to the playback of calls of familiar adult females (Mulard et al., 2010). However, the authors found no difference between the sexes in their response to adult female calls (number of calls and latency of response). Still, the vocal response to a playback and to real vocal interactions is probably different. Also, contrary to this previous study, our adult females were unfamiliar to the juveniles, and this could explain the differences between our results. It thus remains to be tested whether the difference of vocal activity between juvenile males and females in our results is triggered by the sex and/or the familiarity of the adults interacting with the juveniles.

Cross-correlation is a measure of vocal synchrony between individuals. A high cross-correlation between two individuals (two nodes in the acoustic network) means that these individuals usually vocalize together (or remain silent together) within 500 ms. Akin to spatial connectedness, we considered that birds that vocalize regularly together are connected. In our results, the cross-correlation was lower between one juvenile and one adult than between two juveniles, which was itself lower than between two adults. In our setup, all adults were females (no adult male), so interactions between juvenile males and adults could not be vocal imitation for song learning (like with a male song tutor) but could be social reinforcement of song production by adult females. However, more generally, interactions between juveniles (females or males) and adults could be social reinforcement of vocalization use. In our results, interactions between juveniles and adults showed less synchrony than vocal interactions between juveniles, so the latter probably function as stronger reinforcements of vocalization use. In our study adult females were familiar with each other and not with the juveniles, and juveniles were familiar with each other and not with the adult females. These differences in familiarity may therefore have contributed to the lower cross-correlation between adult females and juveniles, as individuals may respond more to familiar individuals. However, cross-correlation and maximum transition values were similar between young adults in the second phase and adult females in the first phase, even though not all young adults were familiar with each other. Furthermore, we did not observe an increase in average cross-correlation or maximum transition values between the first and second recording days per phase, although all four birds were presumably becoming more familiar with each other as they remain together in the same room. Overall, familiarity is therefore unlikely to fully explain our results.

Instead, our results suggest that (1) individuals interact preferentially within their age group (because the cross-correlation between one adult and one juvenile had the lowest value), and that (2) adults are more precise and regular in their vocalization timing (because they had the highest cross-correlation value). Adults may be less likely to interact with a juvenile when juveniles are less reliable in the timing or information content of their vocalizations or when the information juveniles provide is irrelevant for adults. For example, in juvenile Richardson's ground squirrels (*Spermophilus richardsonii*), if an individual frequently calls when no predators are nearby, its calls do not reliably predict the presence of a predator and the calls of this individual are ignored by others. Young individuals may call in response to more stimuli, many of which are not threatening to adults (Cheney and Seyfarth, 1990; Hanson and Cross, 1997), and it might be advantageous for adults to ignore the calls from the juveniles. In a learning context, Chimpanzees (*Pan troglodytes*), are highly specific in their selection of conspecifics as models for observation: in response to a novel item, they watch and learn from the nut-cracking activity of individuals in the same age group or older, but not younger than themselves (Biro et al., 2003).

Our analysis of turn-taking involving Markov chains showed that the probability of having a juvenile vocalization following a juvenile vocalization was higher than any other possibility. Contrary to the cross-correlation, turn-taking does not take into account the delay between vocalizations. Therefore, a high Markov probability between juveniles means that juveniles vocalized preferentially after a juvenile vocalization (without having an adult's vocalization between them), but the delay can be of any value (so potentially above the 500 ms threshold used in the cross-correlation analysis). The respect of turn-taking requires attention and control and may be less easy to achieve for juveniles. Hauser (1992) showed that juvenile Vervets monkeys *(Chlorocebus pygerythrus)* overlap other individuals' calls more often than adults. This study estimated that 1/38 calls were interrupted when the exchange was between adults compared to 6/20 when the interacting individuals were young. This observation suggests that the ability to respect turns may be acquired during development. In Campbell's monkeys (*Cercopithecus campbelli*), the young are 12 times more likely than adults to interrupt turn-taking by vocalizing twice successively. Besides, only adult Campbell's monkeys displayed different levels of interest when hearing playbacks of vocal exchanges respecting or not the turn-taking rule (Lemasson et al., 2011). In nightingales (*Luscinia megarhynchos*), it has been suggested that overlapping (and therefore breaking the turn-taking rule) may be perceived as a directed aggressive signal (Naguib and Kipper, 2005). In this species, alternation in exchanges suggests that turn-taking rules allow turns to be taken between two or more interlocutors, and overlapping elicits “irritation” or a rupture of the exchange.

The cross-correlation between 1 male and 1 female increased from juveniles to young adults, whereas it remained the same between 2 males or 2 females. The young adults had reached the sexual maturity (between 2 and 3 months in zebra finches). In the wild, zebra finch juveniles are fully independent at 35 days and may start forming pairs at 3 months old (Zann, 1996). The tendency to interact with individuals from the opposite sex may increase after sexual maturity. In wild Chacma Baboons (*Papio Cynocephalus Ursinus*), females' reproductive state affects males' tendency to call to them (Palombit et al., 1999). Males grunted more often when approaching estrus females and lactating females, and rarely when approaching pregnant females. In addition, affinitive interactions between 1 male and 1 female occurred significantly more often when males grunted than when they silently approached females.

In this study we decided to keep all vocalizations types together, because we had too many factors interacting to be able to analyze rules of vocalization type use with a sufficient sample size. Besides, among all vocalizations types that zebra finches can produce, in the conditions of our experiment (cages at short distances) only three of them were produced: tets, distance calls, and songs. However, it would be interesting to study the vocal dynamics by separating the different vocalization types, because the dynamic of vocal exchange could change according to call type, as suggested by Gill et al. (2015).

Also, preventing physical contact and free movement of the birds is a limitation. However, our approach has the advantage to control the position of the birds. In a recent study, devices mounted on the birds were used to assign vocalizations in freely moving individuals (Gill et al., 2015) but it did not give the spatial position of each bird. New technologies are needed to be able to control for these different aspects at the same time.

Taken together, our results suggest that juveniles and adults have a separate vocal network (i.e., same age class individuals form distinct connected components within the network), and juveniles integrate the properties of the adult vocal network during ontogeny. Our findings highlight the benefits of considering acoustic networks, beside spatial associations, to infer social interactions within groups.

## Ethic statement

Experiments were performed under the authorization no. 42-218-0901-38 SV 09 (ENES Laboratory, Direction Departementale des Services Veterinaires de la Loire) and were in agreement with French and European legislation regarding experiments on animals.

## Author contributions

MF carried out the data extraction and analysis, statistical analyses, drafted the manuscript. HS participated in the data analysis and drafted the manuscript. CV and MM designed the study, coordinated the study, performed the recordings and drafted the manuscript; All authors gave final approval for publication.

### Conflict of interest statement

The authors declare that the research was conducted in the absence of any commercial or financial relationships that could be construed as a potential conflict of interest.

## Supplementary material

Supplementary Figure 1 **Females' number of vocalizations from juvenile to young adult**. Red points with red bars are mean ± *SE* values on all female individuals (*N* = 33). Black points with black bars are mean ± *SE* values on each female for all recordings, when the number of vocalizations decreased from juvenile to young adult. Gray points with gray bars are mean ± *SE* values on each female for all recordings, when the number of vocalizations increased. Lines connect paired values from same individuals.

Supplementary Figure 2 **Males' number of vocalizations from juvenile to young adult**. Red points with red bars are mean ± *SE* values on all male individuals (*N* = 33). Black points with black bars are mean ± *SE* values on each male for all recordings, when the number of vocalizations decreased from juvenile to young adult. Gray points with gray bars are mean ± *SE* values on each male for all recordings, when the number of vocalizations increased. Lines connect paired values from same individuals.

Supplementary Figure 3 **Acoustic networks of four groups with juveniles**. Nodes are individuals (J_i_ states for juvenile i and Ad_i_ states for adult i). Edges thickness is an affine function of the average vocal cross-correlation on all recording days for each dyad.
