# Supplementary figures and images for: A New Semi-automated Method for Assessing Avian Acoustic Networks Reveals that Juvenile and Adult Zebra Finches Have Separate Calling Networks
Source: Front Psychol. 2016 Nov 29;7:1816. doi: 10.3389/fpsyg.2016.01816 (PMC5126114; doi:10.3389/fpsyg.2016.01816)

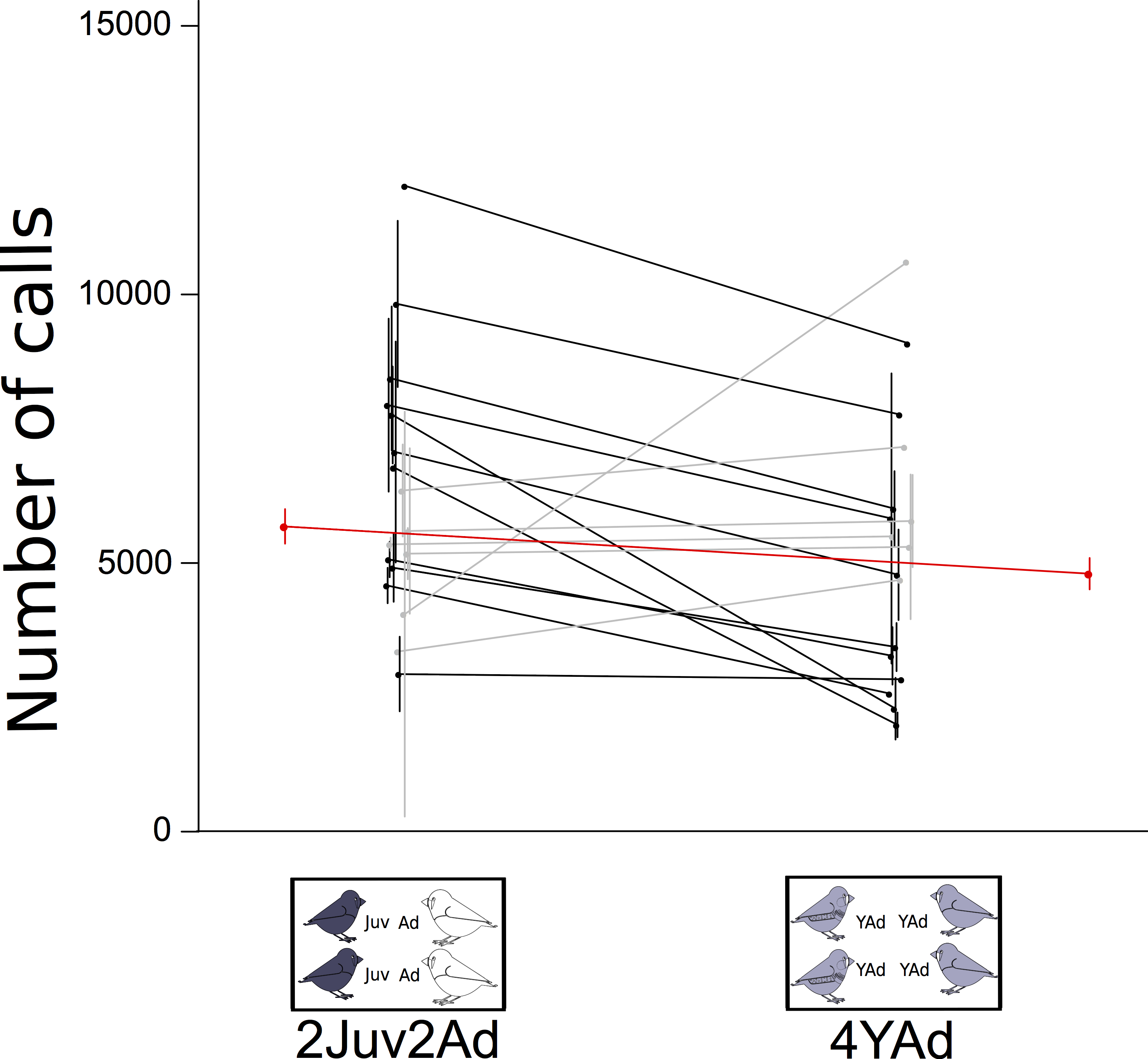

Supplement: Supplementary Figure 1 — Females' number of vocalizations from juvenile to young adult. Red points with red bars are mean ± SE values on all female individuals (N = 33). Black points with black bars are mean ± SE values on each female for all recordings, when the number of vocalizations decreased from juvenile to young adult. Gray points with gray bars are mean ± SE values on each female for all recordings, when the number of vocalizations increased. Lines connect paired values from same individuals. [file Image1.JPEG]

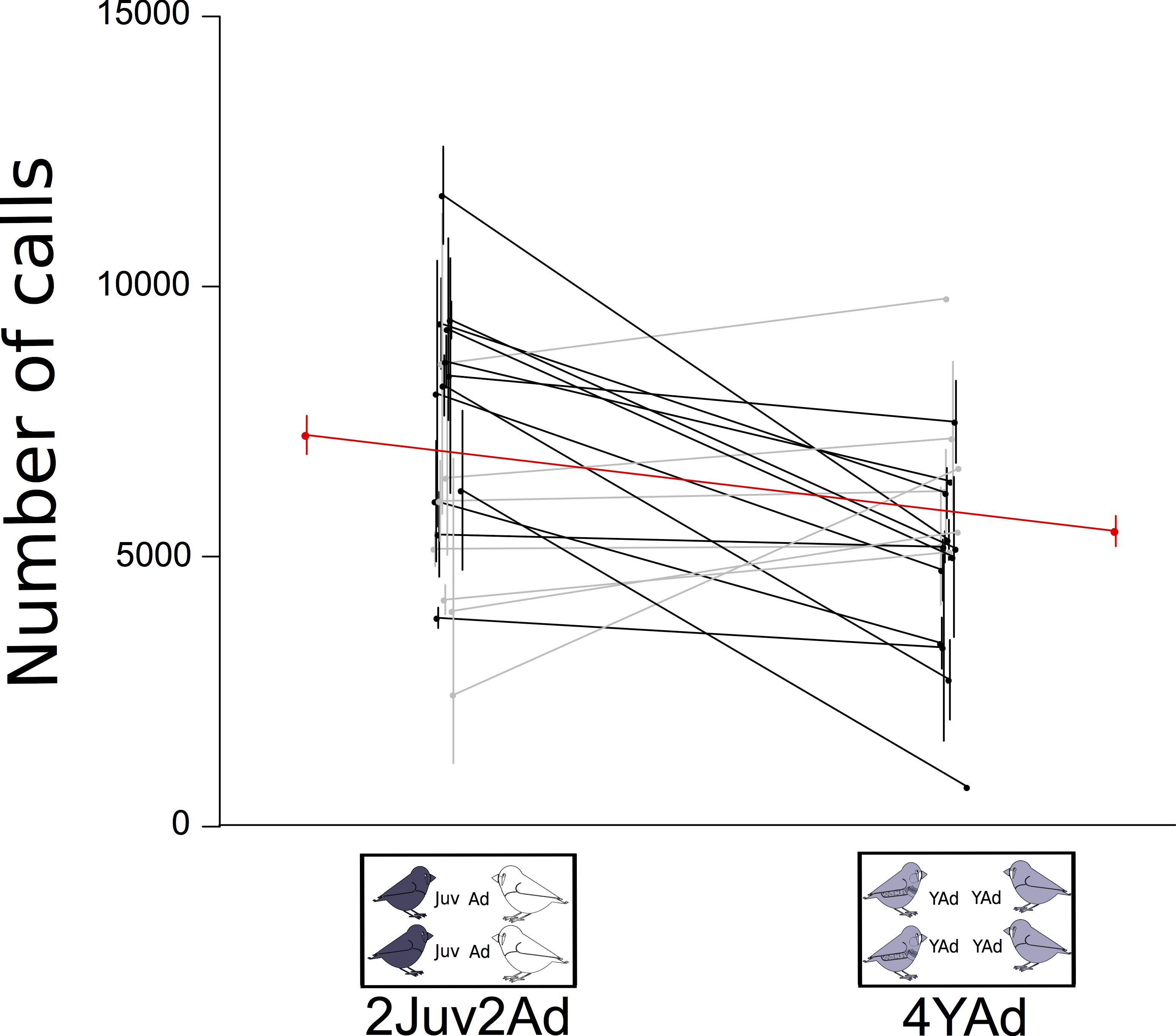

Supplement: Supplementary Figure 2 — Males' number of vocalizations from juvenile to young adult. Red points with red bars are mean ± SE values on all male individuals (N = 33). Black points with black bars are mean ± SE values on each male for all recordings, when the number of vocalizations decreased from juvenile to young adult. Gray points with gray bars are mean ± SE values on each male for all recordings, when the number of vocalizations increased. Lines connect paired values from same individuals. [file Image2.JPEG]

● juvenile  
○ adult

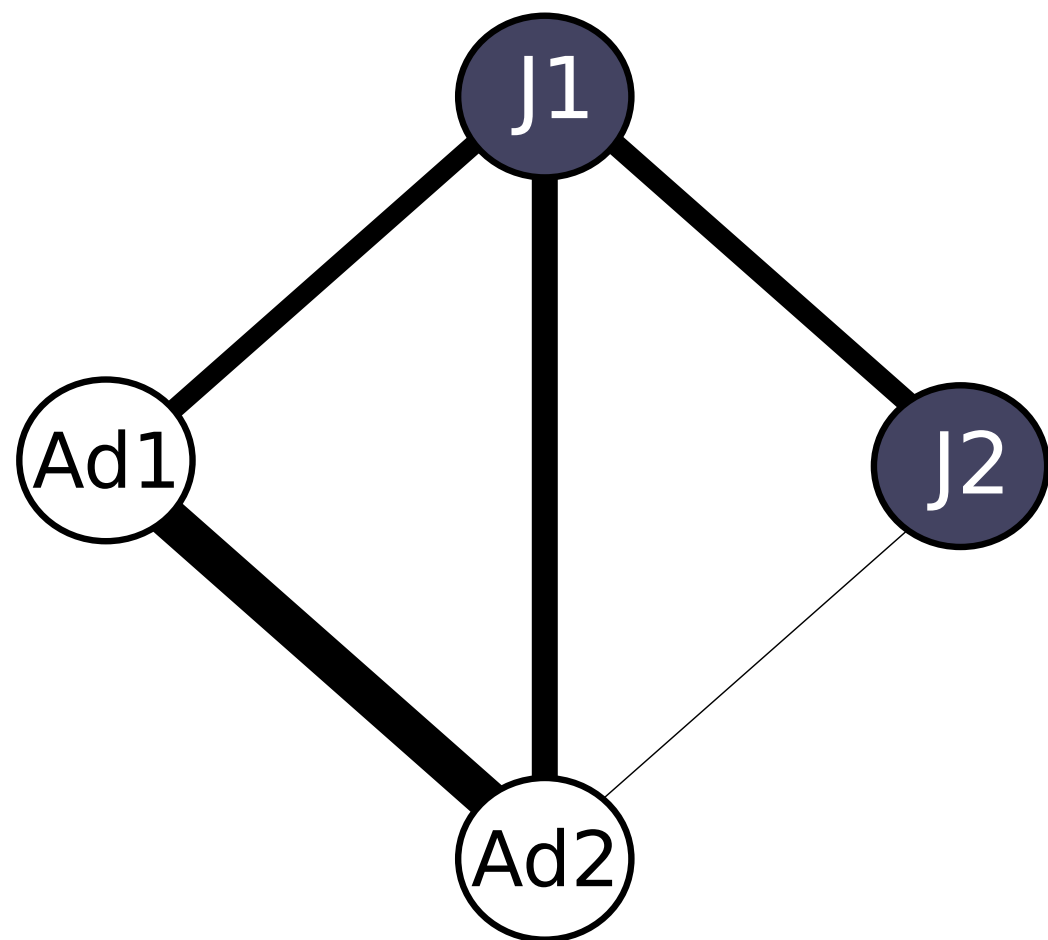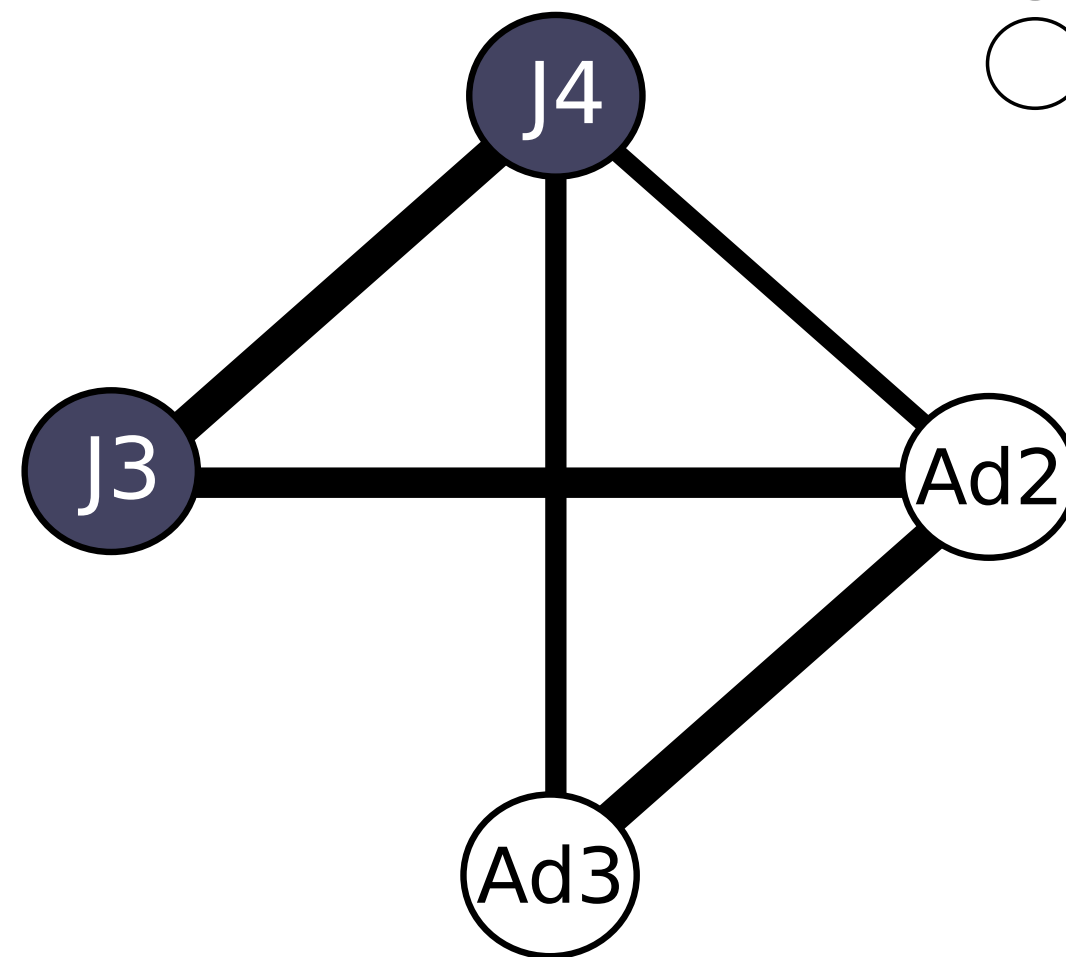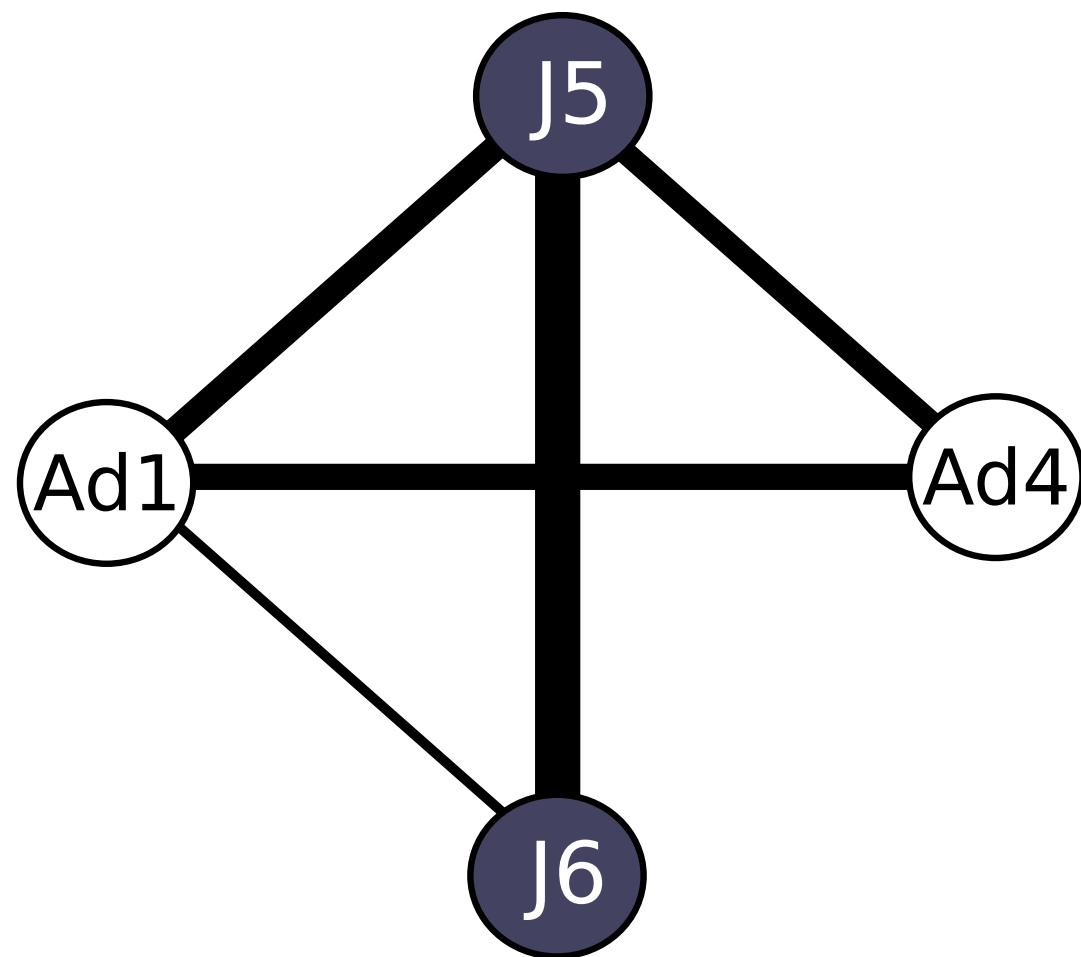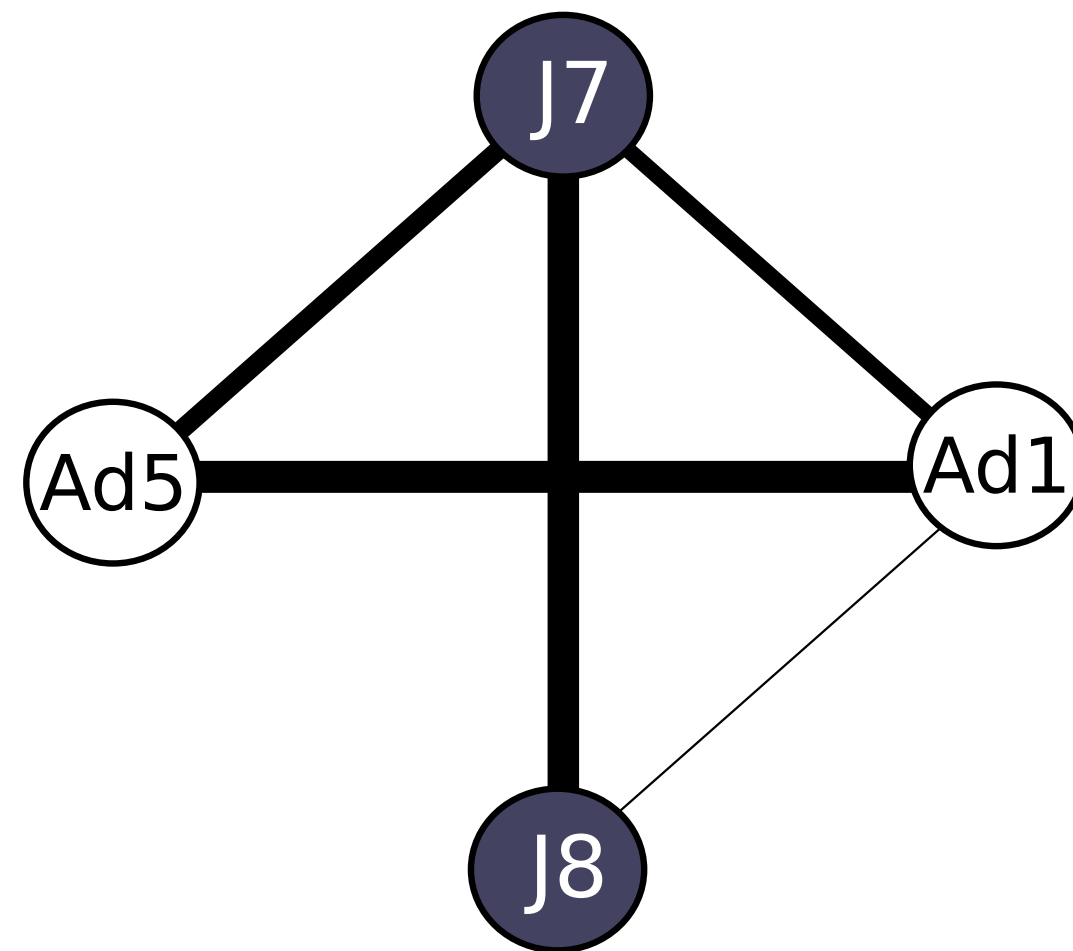

Supplement: Supplementary Figure 3 — Acoustic networks of four groups with juveniles. Nodes are individuals (Ji states for juvenile i and Adi states for adult i). Edges thickness is an affine function of the average vocal cross-correlation on all recording days for each dyad. [file Image3.PDF]
